# Gum Arabic Edible Coating Reduces Postharvest Decay and Alleviates Nutritional Quality Deterioration of Ponkan Fruit During Cold Storage

**DOI:** 10.3389/fnut.2021.717596

**Published:** 2021-10-18

**Authors:** Qiang Huang, Chunpeng Wan, Yajie Zhang, Chuying Chen, Jinyin Chen

**Affiliations:** Jiangxi Key Laboratory for Postharvest Technology and Nondestructive Testing of Fruits & Vegetables, College of Agronomy, Jiangxi Agricultural University, Nanchang, China

**Keywords:** Ponkan fruit, gum arabic coating, nutritional quality, antioxidant enzymes, postharvest cold storage

## Abstract

The storability recession during storage limits the postharvest storage life of Ponkan (*Citrus reticulata* Blanco cv. Ponkan) fruit and its nutritional value, which potentially lead to huge losses. To develop an effective technique to reduce Ponkan fruit postharvest decay and to maintain the nutritional quality, the preservation effect of 9, 12, and 15% postharvest gum arabic (GA) coatings on Ponkan fruit was investigated. The 12 and 15% GA coatings effectively reduced fruit decay as well as weight loss, retained higher total soluble solids (TSS) content, suppressed titratable acidity (TA) degradation, and postponed the rise in ripening index (RI). Moreover, the 12% GA-coated fruit exhibited a lower respiration rate, electrical conductivity, and malondialdehyde (MDA) accumulation than the uncoated (control) fruit. The 12% GA coating treatment decreased the loss of ascorbic acid (AsA), total phenols, and total flavonoids and maintained higher amounts of non-enzymatic antioxidants. Furthermore, the 12% GA coating treatment increased antioxidant enzymes' activities as well as delayed the reduction of total antioxidant capacity (TAC). These results suggest that, with the cold storage increasing time, the 12% GA-coated fruit exhibited better postharvest storability and higher nutritional quality than the control fruit. The GA coating treatment could be used as a commercial wax to improve postharvest storability, extend its storage life, and maintain the nutritional value of Ponkan fruit up to 120 days of cold storage.

## Introduction

Ponkan (*Citrus reticulata* Blanco) is a widely cultivated and consumed fruit in China owing to its rich juice, delicious taste, easy to peel, high yield, and affluent nutrients, such as vitamin C [VC, or ascorbic acid (AsA)], flavonoids, and other antioxidants (1, 2). However, Ponkan fruit is easily subjected to heavy losses during harvest, storage, transportation, and marketing, which has brought a tremendous threat to its industry. Although there are various causes for an increase in economic losses after harvest, the most important ones are the postharvest diseases caused by fungal infection as well as physiological damage (nutritional quality deterioration, oil spotting/oleocellosis, granulation, etc.) (3–6).

There is considerable evidence that the imbalance of reactive oxygen species (ROS) metabolism may result in irreversible damage to the cytoplasmic membrane and mitochondria due to excessive ROS production and shortens the storage life of fresh horticultural fruits, such as orange (7, 8), pummelo (9), strawberry (10), and tomato (11). Furthermore, harvested fruits have a functional ROS-scavenging system that includes ROS-scavenging enzymes [e.g., superoxide dismutase (SOD), catalase (CAT), peroxidase (POD), ascorbate peroxidase (APX), and glutathione reductase (GR)], along with antioxidant substances, such as AsA, phenolics, and flavonoids (9, 12). The efficiency of a ROS-scavenging system in plant cells is a critical factor in the tolerance to oxidative stress and enhancing the storability in harvested fruits. It is well-known that the pre-storage application of salicylic acid treatment can effectively reduce the loss of postharvest decay and increase AsA content, total phenolic contents (TPCs), antioxidant capacity, as well as antioxidant enzyme activities in harvested “Kinnow” mandarins during storage (8). Nie et al. (9) found that pummelo fruits exhibited a poor antioxidant capacity as a direct consequence of low nutritional quality; these fruits were highly susceptible to senescence stress and deviated from the acceptability of consumers by exhibiting high juice sac granulation. Therefore, the activation of an antioxidant defense system could be a promising strategy to stimulate the resistance of fruits and to prolong their storage life.

To reduce citrus fruit decay and minimize the quality deterioration during postharvest storage, the pre-storage application of semipermeable edible films has been shown to protect the fruit from fungal attacks and to reduce postharvest nutrient decomposition from fruit respiration. Various edible coating films from polysaccharides have been studied for citrus fruit postharvest preservation. For example, the edible coating formulations based on *Aloe vera* gel (13), pea starch and guar gum (14), Persian gum (7), wax coating (15), carnauba wax (16), methylcellulose, carboxymethyl cellulose (CMC) and chitosan (6, 17), hydroxypropyl methylcellulose (18), and sodium alginate (1.5%) with the *Ficus hirta* fruit extract (19), maintained fruit quality and extended postharvest storage-life of citrus fruit. Among the potential edible coatings, gum arabic (GA) could be a natural polysaccharide-based edible coating for the postharvest preservation of horticultural fruits.

As a novel water-soluble polysaccharide, GA is derived from the gum exudates of *Acacia senegal* tree and mainly constituted of D-galactose, L-arabinose, L-rhamnose, and D-glucoronic acid with a handful of proteins or metal ions (20, 21). GA is broadly used as a natural film preservative for its water solubility, film forming, antioxidant activity, and emulsification (22, 23). Earlier studies have proven that GA coating alone or enriched with other preservative agents reduced postharvest decay and maintained the overall quality of harvested climacteric fruits such as banana, guava, mango, and tomato (11, 24–27). However, there is no report of the pre-storage treatment of GA coating on the postharvest decay and overall quality deterioration of citrus fruits. Thus, the aim of this study was to explore the effect of GA coating on the quality characteristics (e.g., decay rate, weight loss, nutrient content, antioxidant capacity, and enzyme activity) of Ponkan fruit during cold storage at 10°C.

## Materials and Methods

### Fruit Materials

Ponkan fruits used in this experiment were freshly harvested at the commercial maturity stage [size: 140–160 g; total soluble solids (TSS) content: 10.6–11.2%; titratable acid (TA) content: 1.46–1.52%, and citrus color index (CCI): 4.83–5.46] in Jing'an city (28°48′47″ N and 115°23′59″ E, Jiangxi Province, China), were selected based on a uniform size (136–160 g per fruit) and shape and on the lack of visual mechanical injuries or pathogen infections, and then transported immediately to our laboratory within 3 h.

### Preparation of GA Solutions and Coating Treatment

Gum arabic powder (CAS: 9000-01-5) was purchased from Sigma-Aldrich Chemical Co., St. Louis, MO, USA. Briefly, the three concentrations (9, 12, and 15%, *w/v*) of GA coating solutions were prepared by dissolving 45, 60, and 75 g of GA powder in 500 ml of sterile water and by heating at 40°C in a hot plate for 60 min. After cooling to room temperature, 5 g of glycerol was added as a plasticizer to ameliorate the flexibility of GA coating solutions.

After pre-storage for 3 days, 2,400 healthy fruits without mechanical injury were washed in running water, air-dried, and randomly separated into four groups. For the three coated groups, 600 fruits of each group were dipped in each concentration of GA coating solutions (9, 12, and 15%) for 2 min. The remaining 600 fruits without GA coating being termed as the control group. Following air-drying, all the coated and control groups were placed into plastic crates (overall dimension: 48 cm × 32 cm × 25 cm), enclosed with a low-density polythene bag (*d* = 0.04 mm) to create a high humidity (~95%) condition, and then stored at 10°C for 120 days. For every 15 days during postharvest storage at 10°C, three replications were performed in each treatment and every replication contained 10 fruits to measure their biochemical parameters.

### Fruit Decay and Weight Loss

The incidence of the decayed Ponkan fruit showing the presence of fungal rot symptom during cold storage at 10°C was measured to be similar to that of the 300 fruits of three replicates in each group and was calculated on the initial fruit number for each group every 15 days, and is expressed in percentage (%).

The weight loss of Ponkan fruit was determined to be similar to that of the 30 fruits of three replicates in each group, and the calculation of weight loss was as follows: (the initial fruit weight − the weight of the stored fruit)/the initial fruit weight × 100.

### TSS, TA, and Ripening Index

About 10.0 g of the fruit pulp from 10 fruits per replicate in each group was homogenized and centrifuged at 943,300 × g for 15 min. The supernatant was obtained and used for measuring the contents of TSS and TA. TSS content was measured using the aid of a handle sugar tester (RA-250WE, Atago, Tokyo, Japan), and the result was expressed in %. TA content was assayed by titration with 0.1 M sodium hydroxide and expressed in % of citric acid. Ripening index (RI) was calculated by dividing TSS by TA (28).

### Respiration Rate, Electrolyte Leakage, and Malondialdehyde Content

About 10 fruits were weighted for determining the respiration rate according to the method of Chen et al. (19, 29) using a GXH-3051H IR carbon dioxide (CO_2_) fruit respiration determinator, and the respiration rate was expressed as the production of CO_2_ on a fresh weight (FW) basis per hour (mg CO_2_ kg^−1^ h^−1^).

To estimate the integrity of the cell membrane, electrolyte leakage was assayed as previously reported by Chen et al. (6). About 20 10-mm-diameter discs from 10 fruits were rinsed in 40 ml of deionized water and then shaken at 25°C. After 30 min, the initial electrolyte leakage was measured with the aid of a DDS-307A conductivity meter (Rex, Shanghai, China). Then, the above solution was boiled for 20 min and cooled down to 25°C, and the final electrolyte leakage was measured. Electrolyte leakage was expressed in % and calculated by dividing the initial value by the final value.

Malondialdehyde (MDA) content was determined based on the thiobarbituric acid (TBA) method as represented by Nie et al. (9). Peel samples (5.0 g) from 10 fruits were adequately homogenized with 25 ml of pre-cooled 50 mM phosphate buffer [pH 7.8, containing 0.5 mM ethylenediaminetetraacetic acid (EDTA) and 2% (*w/v*) polyvinylpyrrolidone (PVP)] and then centrifuged at 12,000 × g for 20 min at 4°C. The supernatant (2 ml) was mixed with an equal amount of 0.5% (*w/v*) TBA, boiled for 30 min, and then cooled to room temperature. After being centrifuged at 6,000 × *g* for 10 min, the absorbances of the supernatant were measured at 450, 532, and 600 nm, respectively. MDA content was expressed in mmol g^−1^ FW.

### AsA Content, TPC, Total Flavonoid Content, and Total Antioxidant Capacity

The content of AsA was quantified as described by Chen et al. (9, 19) with slight modifications. Briefly, 5.0 g of fruit juice from a 10-fruit mixture was mixed with 50 ml of 2% (*w/v*) oxalic acid solution. After the extraction for 10 min, AsA content was monitored by the titration method of 2, 6-dichlorophenol indophenol (DPIP) and was expressed in mg 100 g^−1^ based on FW.

Following the Folin–Ciocalteu method reported by Khorram et al. (7) and the method described by Sarker et al. (30), both TPC and total flavonoid content (TFC) were assayed, by using GA and rutin as the standard curve, respectively, and were expressed in mg g^−1^ FW.

Total antioxidant capacity (TAC) in Ponkan fruit was determined by using 2, 2-diphenyl-1-picrylhydrazyl (DPPH) radical assay as described by Luo et al. (31) with slight revisions. A mixture of 1.5 ml of the extracted juice and 1.5 ml of DPPH (0.2 mM) solution (prepared with 95% ethanol) was held in the dark for 30 min at 25°C before the absorbance at 517 nm was read by a TU-1950 spectrophotometer (Beijing Persee Co. Ltd., Beijing, China). TAC for scavenging DPPH radical was calculated as [(control OD_517_ - sample OD_517_)/control OD_517_] × 100, and the result was expressed in %.

### Assays of Antioxidant Enzyme Activities

Crude enzymes of Ponkan fruit were extracted using the method of Nie et al. (9) by homogenizing 2.0 g of the frozen peel powder (ground in a Retsch MM 400 grinder) with 10 ml of pre-cooled 50 mM phosphate buffer (pH 7.5, containing 0.5 mM AsA, 1 mM EDTA, and 2% PVP) and by centrifuging at 12,000 × g at 4°C for 30 min. The supernatant was gathered for assaying the activities of SOD, CAT, POD, and APX.

The SOD activity was measured according to the hydroxylamine method by applying with a SOD test kit (No: A001-1-2; Nanjing Jiancheng, Jiangsu, China). One unit of SOD activity was determined to inhibit 50% of nitroblue tetrazolium (NBT) photoreduction per minute (29). The activities of CAT, POD, and APX were determined by using the method of hydrogen peroxide (H_2_O_2_) decomposition as given in Nakano and Asada (32). One unit of CAT, POD, and APX activities was defined as an increment of 0.01/min in the absorbance of the reaction solution at 240, 470, and 290 nm, respectively. The results of the abovementioned enzyme activities were expressed in U g^−1^.

### Statistical Analysis

All data of quantitative parameters were subjected to a one-way ANOVA using the SPSS software (Version 17.0) with treatment and storage time as sources of variation and were expressed as the mean plus SD from the three biological replications. The level of significant difference was compared by Duncan's multiple range tests at *p* < 0.05.

## Results and Discussion

### Effects of GA Coating on Fruit Decay, Weight Loss, Quality Attributes, and RI

Fruit rot decay is an important reason for the postharvest quality deterioration of harvested citrus fruit (8, 33). The decay rate of the stored Ponkan fruit increased with the prolongation of storage time and reached its maximum at 120 days of cold storage (Table 1). The postharvest decay rate was significantly lower (*p* < 0.05) in the 9, 12, and 15% GA-coated group in comparison with the uncoated control group (Table 1). At the termination of the storage, the uncoated control group showed a 17.67% ± 1.53% loss in decay rot, whereas the decay rate of the 9, 12, and 15% GA-coated Ponkan fruit was 13.67% ± 1.15%, 9.67% ± 1.15%, and 11.33% ± 1.53%, respectively. Numerous studies have been demonstrated that GA coating had no potential in controlling postharvest fungal diseases in horticultural products (34, 35). Maqbool et al. found that 10% GA coating had no effect on controlling anthracnose, the causal agent of *Colletotrichum musae* in banana and *Colletotrichum gloeosporioides* in papaya (35). Our result suggested that the lowest decay rate was observed in the 12% GA-coated group, indicating that GA coating has a preventive effect in reducing fungal infection. Owing to its ability to reduce the attacks from pathogenic fungi, such as *Penicillium digitatum, Geotrichum citri-aurantii*, and *C. gloeosporioides*, citrus fruit postharvest decay rot consequently reduced. Similar reports were observed in mango (27), strawberry (10), bell pepper (36) when treated with GA coating solution.

**Table 1:** Variation in postharvest decay rate, weight loss, TSS content, TA content and ripening index of harvested Ponkan fruit treated with different GA coatings.

**Quality attribute**	**Treatment**	**Storage period (days)**								
		**0**	**15**	**30**	**45**	**60**	**75**	**90**	**105**	**120**
Decay rate (%)	Control	0 ± 0.00	0 ± 0.00^a^	0 ± 0.00^a^	1.67 ± 0.58^ab^	4.33 ± 0.58^cd^	6.67 ± 1.15^ef^	9.33 ± 0.58^g^	12.33 ± 1.15^hi^	17.67 ± 1.53^j^
	9% GA coating		0 ± 0.00^a^	0 ± 0.00^a^	0.67 ± 0.58^a^	1.67 ± 1.15^ab^	3.67 ± 1.15^c^	6.00 ± 1.00^e^	9.33 ± 1.53^g^	13.67 ± 1.15^i^
	12% GA coating		0 ± 0.00^a^	0 ± 0.00^a^	0 ± 0.00^a^	0.67 ± 0.58^a^	1.67 ± 0.58^ab^	3.67 ± 0.58^c^	6.00 ± 1.00^e^	9.67 ± 1.15^g^
	15% GA coating		0 ± 0.00^a^	0 ± 0.00^a^	0 ± 0.00^a^	1.33 ± 0.58^a^	3.00 ± 1.00^bc^	5.67 ± 1.53^de^	7.67 ± 1.53^f^	11.33 ± 1.53^h^
Weight loss (%)	Control	0 ± 0.00	0.91 ± 0.13^bc^	1.68 ± 0.10^e^	2.36 ± 0.12^g^	3.05 ± 0.18^i^	3.87 ± 0.16^k^	4.55 ± 0.13^m^	5.43 ± 0.16°	6.24 ± 0.14^p^
	9% GA coating		0.72 ± 0.05^ab^	1.30 ± 0.03^d^	1.88 ± 0.11^f^	2.35 ± 0.13^g^	3.05 ± 0.16^i^	3.65 ± 0.08^j^	4.16 ± 0.09^lm^	4.68 ± 0.07^n^
	12% GA coating		0.63 ± 0.11^a^	0.98 ± 0.18^c^	1.50 ± 0.09^de^	2.05 ± 0.08^f^	2.65 ± 0.13^h^	3.25 ± 0.13^i^	3.77 ± 0.05^jk^	4.18 ± 0.12^m^
	15% GA coating		0.58 ± 0.15^a^	0.98 ± 0.17^c^	1.46 ± 0.07^d^	1.93 ± 0.09^f^	2.55 ± 0.09^gh^	3.12 ± 0.03^i^	3.65 ± 0.13^j^	3.96 ± 0.14^kl^
TSS content (%)	Control	10.60 ± 0.10	11.27 ± 0.15^e^	11.70 ± 0.10^h^	12.33 ± 0.15^j^	12.93 ± 0.1^m^	11.90 ± 0.10^h^	11.17 ± 0.06^de^	10.67 ± 0.12^b^	10.37 ± 0.12^a^
	9% GA coating		10.97 ± 0.12^cd^	11.47 ± 0.15^f^	11.93 ± 0.15^hi^	12.27 ± 0.12^ij^	12.70 ± 0.10^l^	12.10 ± 0.10^i^	11.47 ± 0.12^e^	11.17 ± 0.06^de^
	12% GA coating		10.87 ± 0.15^c^	11.23 ± 0.06^e^	11.87 ± 0.06^h^	11.97 ± 0.12^hi^	12.53 ± 0.12^kl^	12.37 ± 0.12^jk^	11.83 ± 0.12^h^	11.53 ± 0.06^fg^
	15% GA coating		10.87 ± 0.06^c^	11.20 ± 0.10^e^	11.73 ± 0.15^h^	11.83 ± 0.12^h^	12.23 ± 0.06^ij^	11.67 ± 0.12^fg^	11.20 ± 0.10^e^	11.13 ± 0.15^de^
TA content (%)	Control	1.49 ± 0.03	1.30 ± 0.05^k^	1.12 ± 0.04^i^	0.92 ± 0.04^g^	0.80 ± 0.04^f^	0.71 ± 0.02^de^	0.66 ± 0.01^cd^	0.59 ± 0.02^b^	0.52 ± 0.03^a^
	9% GA coating		1.38 ± 0.10^l^	1.20 ± 0.06^j^	1.03 ± 0.05^h^	0.90 ± 0.03^g^	0.80 ± 0.03^f^	0.74 ± 0.02^e^	0.66 ± 0.02^cd^	0.63 ± 0.03^bc^
	12% GA coating		1.40 ± 0.02^lm^	1.27 ± 0.03^k^	1.15 ± 0.03^ij^	1.01 ± 0.04^h^	0.90 ± 0.05^g^	0.82 ± 0.02^f^	0.74 ± 0.03^e^	0.71 ± 0.02^de^
	15% GA coating		1.44 ± 0.02^m^	1.29 ± 0.05^k^	1.20 ± 0.04^j^	1.02 ± 0.04^h^	0.94 ± 0.04^g^	0.83 ± 0.03^f^	0.75 ± 0.02^e^	0.71 ± 0.02^de^
Ripening index	Control	7.12 ± 0.10	8.66 ± 0.21^b^	10.45 ± 0.30^e^	13.39 ± 0.64^gh^	16.12 ± 0.7^m^	16.68 ± 0.59^mn^	17.51 ± 0.69^no^	18.01 ± 0.59^n^	20.13 ± 1.06°
	9% GA coating		7.96 ± 0.09^ab^	9.54 ± 0.32^cd^	11.61 ± 0.60^f^	13.65 ± 0.47^gh^	15.86 ± 0.63^lm^	16.47 ± 0.48^m^	17.42 ± 0.28^no^	17.86 ± 0.68^n^
	12% GA coating		7.74 ± 0.09^a^	8.83 ± 0.17^bc^	10.32 ± 0.19^de^	11.84 ± 0.29^f^	14.02 ± 0.61^hi^	14.77 ± 0.39^jk^	15.99 ± 0.60^lm^	16.35 ± 0.38^m^
	15% GA coating		7.52 ± 0.11^a^	8.70 ± 0.29^bc^	9.80 ± 0.40^de^	11.67 ± 0.43^f^	13.06 ± 0.56^g^	14.36 ± 0.43^ij^	15.18 ± 0.57^kl^	15.82 ± 0.47^lm^

*n = 3 for decay rate, weight loss, TSS content, TA content and ripening index (mean ± standard deviation of three replications). Means with different lower-case letters indicate significant differences according to Duncan's multiple range tests at P < 0.05*.

Postharvest weight loss is a dissatisfactory phenomenon of harvested citrus fruit due to water evaporation and nutrient degradation and is generally known as a key metric for assessing the postharvest freshness of the fruit and its storability during postharvest storage (28, 37). Weight loss increased during the storage period in all coated and uncoated control groups and reached the maximum at the end of storage (Table 1). The highest weight loss was observed in the uncoated control group at the end of the storage period. The 12 and 15% GA-coated Ponkan fruit presented less weight loss compared with the 9% GA-coated and control fruit. However, no significant difference was found between the 12 and 15% GA-coated Ponkan fruit. This result was in agreement with the findings of previous studies, which showed that weight loss was reduced with GA coating solution in lime (38), blueberry (21), tomato (39), and guava (40). The reduction of weight loss in the GA-coated Ponkan fruit could be that it acts as a barrier on the fruit surface to reduce water evaporation, restrict gas exchange, seal small wounds, and delay nutrient degradation, thereby reducing weight loss and preserving the postharvest quality of Ponkan fruit.

Total soluble solid is an important quality trait that reflects the nutritional quality of harvested fruits (9, 28). Initially, SSC increased gradually to reach its peak and decreased afterward in all samples (Table 1). After 75 days of cold storage, the highest TSS content was found in the 12% GA-coated Ponkan fruit. The application of GA coating significantly delayed the degradation of TSS and was effective in preserving a higher TSS content compared with the control during the later period of cold storage. As these are known, over-ripened fruits have low levels of soluble solids, and a higher TSS content, which showed that the pre-storage treatment of GA coating could delay the ripening of Ponkan fruit during storage. GA coating postponed the decomposition of nutrients, which resulted in a higher TSS content in guava (*Psidium guajava* L.) fruit (25), “Grand Nain” bananas (24), and strawberry (10). A similar trend was reported by Nie et al. (9) who confirmed that a 1.5% chitosan coating was a promising preservative film for maintaining the nutritional quality of pummelo fruit.

Titratable acid of Ponkan fruit is generally interpreted in the form of citric acid percentage present in citrus fruit, which is an important parameter in maintaining fruit quality (28, 41). TA content in the stored Ponkan fruit decreased progressively during the period of cold storage, with lower values in the uncoated than in GA-coated Ponkan fruit (Table 1). After 45 days of storage, the 12 and 15% GA-coated Ponkan fruit exhibited a significantly higher TA content compared to the control fruit. Conversely, there was no conspicuous difference between the above mentioned two GA-coated groups during the entire storage time. The decline of organic acids has led to fruit respiration with the extension of storage periods. This result was highly similar to the published reports, which showed that TA content of Nanfeng mandarin and strawberry fruit decreased with the extension of storage time (19, 42). Obviously, the pre-storage treatment of GA coating was found to be effective in retaining the level of TA content in Ponkan fruit, which was consistent with the findings of Anjum et al. (40) and Etemadipoor et al. (25), who observed that GA coating alone or mixed with plant extracts postponed the disassembly of TA.

The ripening index is the most important parameter that assesses the senescence process of harvested fruits and determines the fruit's storability (17, 28). RI increased in the three coating treatments and the control sample owing to its prolonging storage time. A higher increase was observed in the control fruit although it was significantly delayed in both the 12 and 15% GA-coated Ponkan fruit (Table 1). This effect was mainly due to a lower loss of TA in the GA-coated Ponkan fruit, manifested by a higher TA content since the GA coating effect on TSS content was slightly smaller. In the storage period, RI increased mainly due to the degradation and loss of organic acids. Edible GA coating acts as a fence to delay postharvest ripening and senescence (28). Lower RI in the GA-coated Ponkan fruit demonstrated a positive effect of GA coating treatment on delaying postharvest senescence and maintaining the storability of horticultural fruits after harvesting. Similar results were reported in guava fruit coated with 5% GA enriched with 2% cinnamon essential oil (25), peach coated with *Aloe vera* gel (43), and plum coated with 2% rosehip oil (44).

### Effects of GA Coating on Respiration Rate and Oxidative Damage

**Figure 1:**
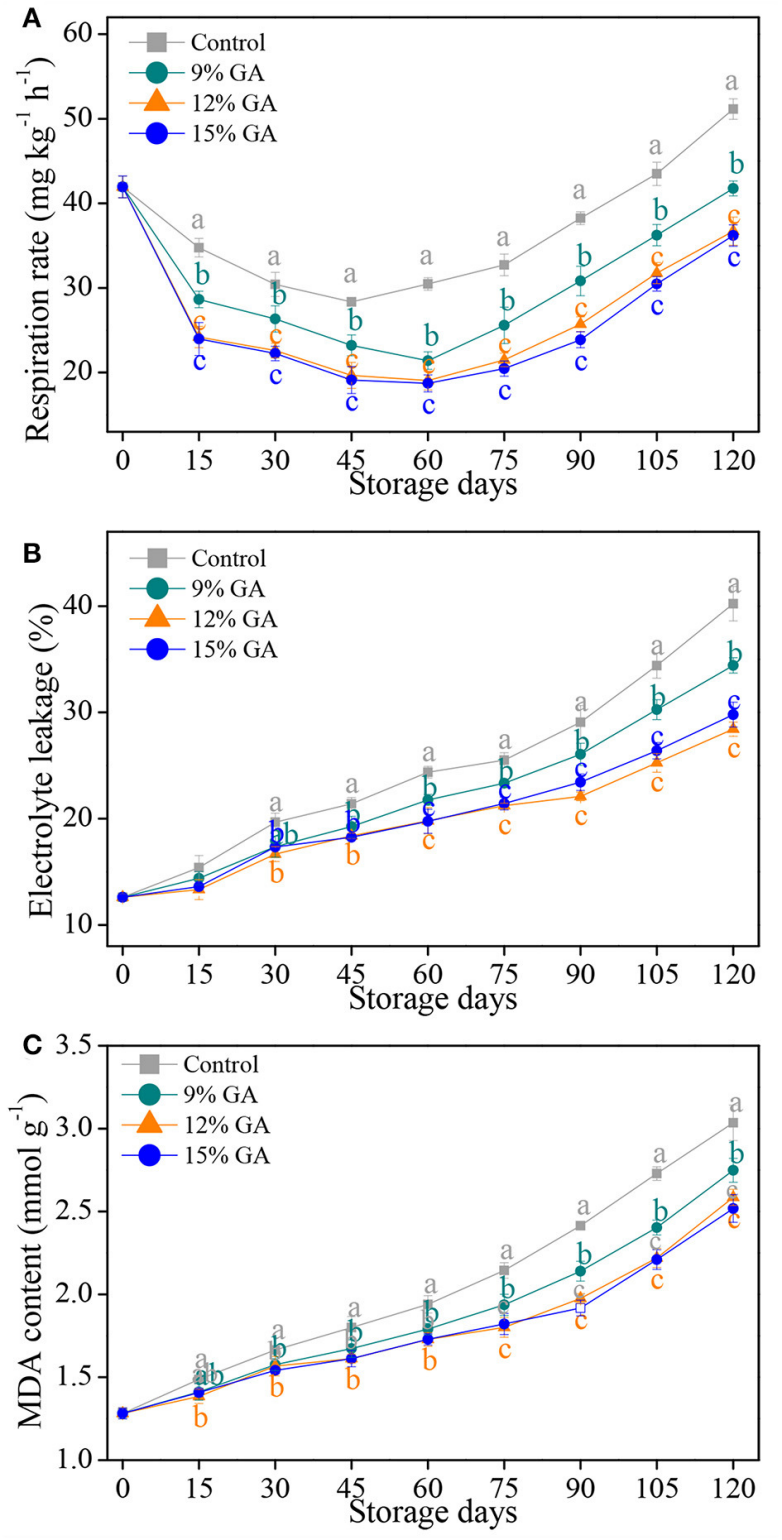
Effects of different gum arabic (GA) coatings on respiration rate **(A)**, electrolyte leakage **(B)**, and malondialdehyde (MDA) content **(C)** in Ponkan fruit stored at 10°C for 120 days. Each value is the mean of the three biological replications. Means labeled with different lowercase letters indicate significant differences (*p* < 0.05) between the control and GA-coated group for every 15 days according to Duncan's multiple range tests.

Fruit respiration is the most basic physiological metabolic activity during its growth, development, and ripening, and its rate is an initial indicator to judge the proper storage-life of horticultural products (14, 28). Respiration rate gradually decreased, initially in the first 45 days of cold storage in both the uncoated control and 12% GA-coated Ponkan fruit, thereafter sharply increased up to the end of cold storage. The 12% GA-coated Ponkan fruit exhibited a lower respiration rate than the control fruit throughout storage (Figure 1A). The prestorage treatment of GA coating inhibited the rate of fruit respiration due to the formation of an edible thin film around the fruit surface, which closed the stomata, inhibited gas exchange, and induced the modification of the internal atmosphere in Ponkan fruit. The results obtained in our study were highly agreed with those of Khorram et al. (7), Khaliq et al. (27), and Ali et al. (11), who all reported that orange, mango, and tomato fruit treated with GA edible coating had reduced the release of CO_2_ and the wastage of oxygen (O_2_), thereby delaying fruit senescence compared to the control.

Electrolyte leakage is used to evaluate the physical injury to the cell membrane resulting from oxidative stress and has been considered as an important indicator for assessing cell membrane permeability (6, 33). An uptrend in the electrolyte leakage of all the samples with increasing storage days is displayed in Figure 1B. Compared to the control Ponkan fruit, the three GA-coated ones exhibited a lower increase in electrolyte leakage. Specifically, at the end of cold storage, the electrolyte leakage of the control fruit increased up to the highest levels, which were 16.9, 41.6, and 35.0% higher than that of the 9, 12, and 15% GA-coated fruit, respectively. Our results implied that the pre-storage treatment of the three GA coatings played an important role in maintaining cell membrane permeability in harvested Ponkan fruit *via* the suppression of electrolyte leakage, which was highly consistent with previous reports, showing that some edible coatings such as GA or chitosan played a positive role in reducing the electrolyte leakage and maintaining the plasma membrane balance in mango and pummelo during postharvest storage (9, 26).

The final product of membrane lipid peroxidation is malondialdehyde, and its accumulation has been seen as an important index for evaluating the level of membrane oxidative damage from plant tissues (29, 31). MDA content in the control and GA-coated Ponkan fruit increased persistently throughout the cold storage of 120 days (Figure 1C). However, the pre-storage treatment of GA coatings slowed down the accumulation of MDA content, and significant differences (*p* < 0.05) were observed among GA coating treatments and storage time considering MDA accumulation. At the end of storage, the lowest MDA content (2.52 mmol g^−1^) was exhibited in the 12% GA-coated fruit, followed by 15% GA coating (2.58 mmol g^−1^), 9% GA coating (2.75 mmol g^−1^), and the highest MDA content (3.03 mmol g^−1^) was recorded in the control fruit, indicating that GA coating treatments could prevent the oxidative damage by lipid peroxidation and maintaining the membrane integrity of Ponkan fruit. The prominent delay in MDA accumulation by 12% GA coating is in agreement with a few reported results (19, 26). Nie et al. (9) also reported a similar result for 1.5% chitosan-coated pummelo (*Citrus grandis* L. Osbeck).

### An Effect of GA Coating on Antioxidant Capacity

**Figure 2:**
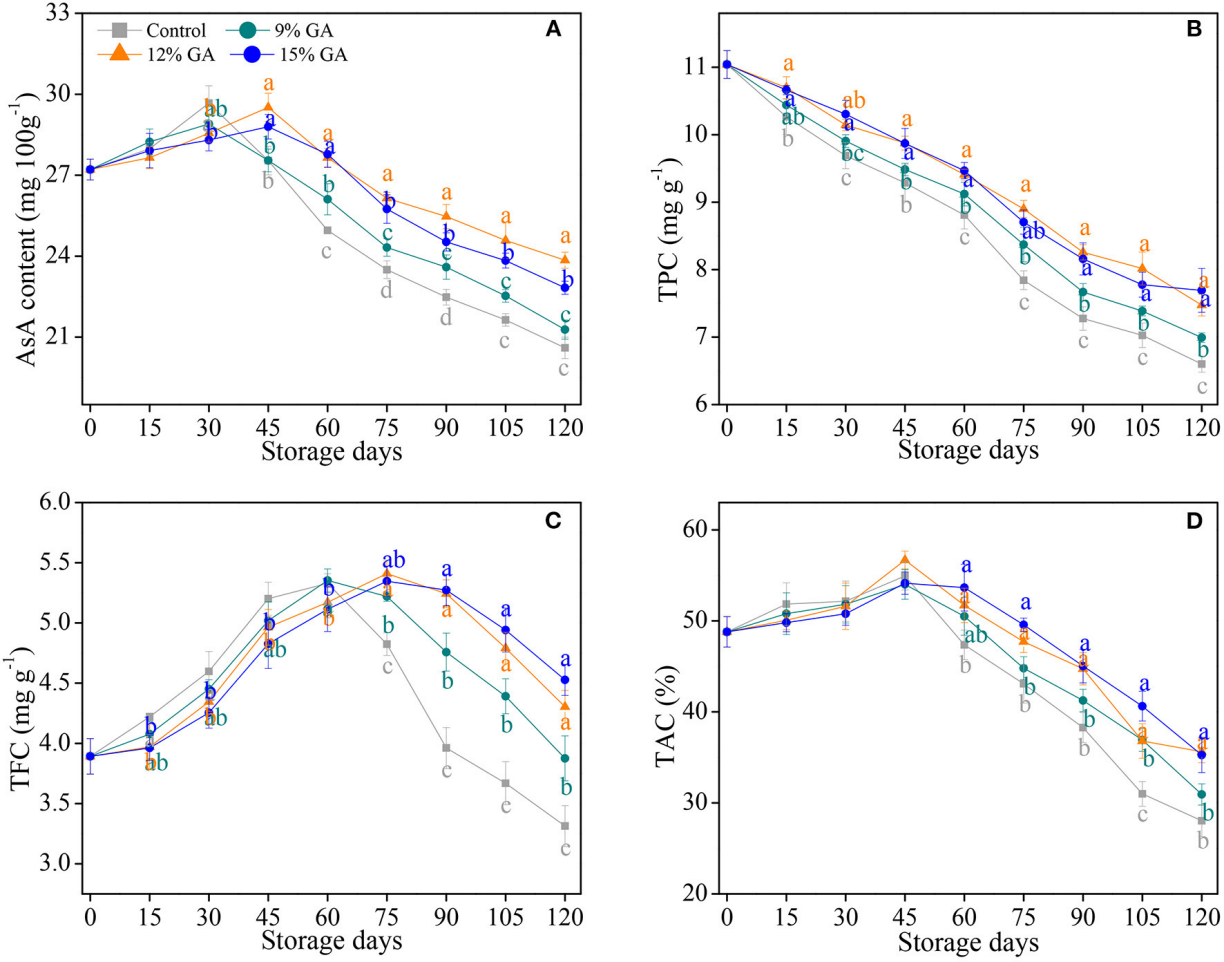
Effects of different GA coatings on ascorbic acid (AsA) content **(A)**, total phenolic content (TPC) **(B)**, total flavonoid content (TFC) **(C)**, and total antioxidant capacity (TAC) **(D)** in Ponkan fruit stored at 10°C for 120 days. Each value is the mean of the three biological replications. Means labeled with different lowercase letters indicate significant differences (*p* < 0.05) between the control and GA-coated group for every 15 days according to Duncan's multiple range tests.

Increasing studies have shown that postharvest film coating maintains the antioxidant capacity of postharvest fruits' response to senescence stress (7, 9, 41, 42, 44). AsA plays a crucial role as a natural antioxidant and reduces the physical injury from oxidative stress (7, 28). As depicted in Figure 2A, AsA content in the control and 9% GA-coated sample increased continuously in the first 30 days of cold storage and dropped constantly in the rest of the storage period, whereas AsA content in the 12 and 15% GA-coated Ponkan fruit peaked at 45 days. In comparison with the 9 and 15% GA-coated group, the 12% GA-coated Ponkan fruit exhibited a higher AsA content after 45 days of storage at 10°C. In the last 30 days of the storage period, the mean AsA contents were maximum and minimum in the 12% GA-coated group and uncoated fruit, respectively. The coating of Ponkan fruit with GA maintained a high level of AsA to reduce oxidation damage. Our results demonstrated the same, as mentioned in previous reports, in which the loss of AsA content was cut down by GA coating in harvested bananas (24), guava (25), and mango (26). GA coating delayed Ponkan fruit postharvest senescence and preserved the nutritional quality by maintaining a higher level of AsA content compared to the control.

Phenolic compounds play a vital role in eliminating free radicals and decrease the development of senescence stress (42, 44). TPC in the control and 12% GA-coated Ponkan fruit gradually decreased with the extension of storage days. The pre-storage treatment of 12% GA coating significantly inhibited a decline of TPC in Ponkan fruit (Figure 2B). At the end of the storage time, Ponkan fruit exposed to 9, 12, and 15% GA coating maintained a higher TPC by 5.9, 13.2, and 16.5% compared to the control sample, respectively. In this study, the TPC of Ponkan fruit was decreased during cold storage at 10°C regardless of pre-storage GA treatments (Figure 2B). However, GA-coated Ponkan fruit showed a higher level of TPC due to the oxidation of phenolics. Nevertheless, a lower level of TPC in the control fruit might be due to the fast degradation of total phenolics (11, 19). It has been well-demonstrated that 10% GA edible coating reserves TPC in Rabbiteye blueberry during the refrigeration storage at 4°C (21). The higher the TPC possessed the more excellent ability to fend off the risks of pathogen infection (19). It might be considered that 12% GA coating reduced the postharvest decay rate in Ponkan fruit *via* the conservation of high amounts of phenolics.

The change of TFC showed a similar trend that of AsA, and the peaks of the control and 12% GA-coated Ponkan fruit were observed at 60 and 75 days, respectively. Pre-storage treatments of 12% and 15% GA coating delayed the peak for 15 days and increased TFC than that in the control during the later period of cold storage (Figure 2C). Meanwhile, the TFC of the 12% and 15% GA-coated group were significantly higher than that of the control group from 75 days of storage (*p* < 0.05). Flavonoids in harvested fruit have the ability to hedge against fungal infection and to positively influence the fruit's storability, and a higher TFC in the 12% GA-coated Ponkan fruit could be related to strengthening the host's disease resistance during the storage condition (19). A sharp decline of TFC after 60 days in the control and 9% GA-coated Ponkan fruit might be due to a higher rate of fruit respiration, which resulted in a greater loss of TFC due to the degradation of certain flavonoids and also the impairment and breakdown of storability, as observed in a previous study on “Hindi-Besennara” mango using an ethanolic extract of propolis (33). Consistent with our results, Alali et al. (24) also observed a high TFC in “Grand Nain” bananas exposed to GA coatings. It was noted that the pre-storage treatment of 12% GA coating postponed the postharvest nutrition deterioration in Ponkan fruit through the irritation of total flavonoids.

As shown in Figure 2D, TAC in the uncoated control and GA-coated fruit increased slightly and peaked at 45 days of cold storage, followed by a sharp decline. However, TAC in the 12 and 15% GA-coated Ponkan fruit was significantly higher in comparison with the control and 9% GA-coated fruit during the last 60 days of cold storage, indicating that the pre-storage application of the GA coating treatment can delay the decrease of TAC. In general, a positive correlation has been reported between antioxidant amounts and TAC. The TAC is highly dependent on fruit ripening and senescence processes. In these processes, the rise and decline of TAC are mainly due to the biosynthesis, accumulation, and degradation of antioxidant components (9, 44). A previous study revealed that GA coating could be applied as an effective means to maintain a higher TAC than the control in blueberry, mango, and guava (21, 26, 40).

### Effects of GA Coating on Antioxidant Enzyme Activities

**Figure 3:**
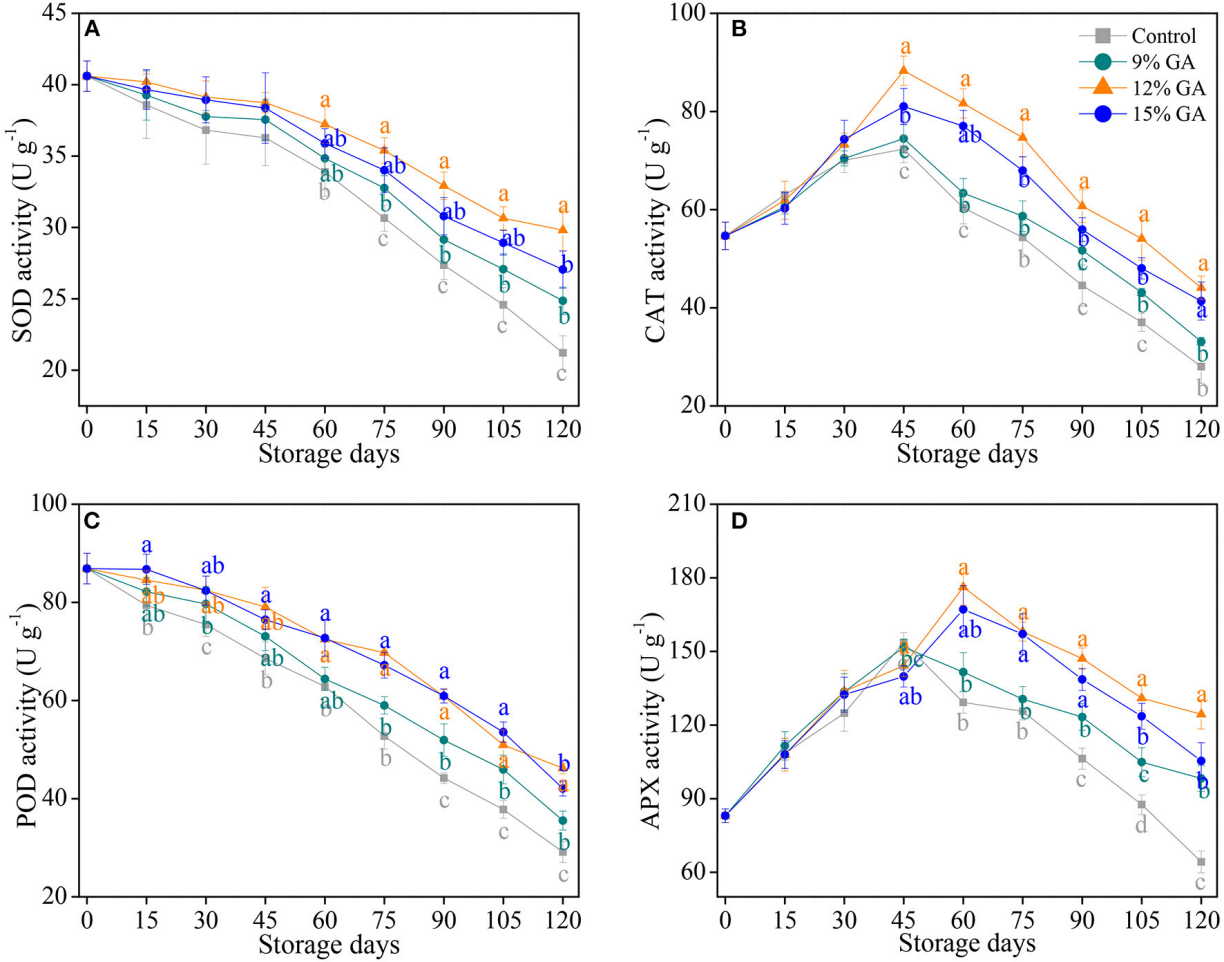
Effects of different GA coatings on superoxide dismutase SOD **(A)**, catalase (CAT) **(B)**, peroxidase (POD) **(C)**, and ascorbate peroxidase (APX) **(D)** activities in Ponkan fruit stored at 10°C for 120 days. Each value is the mean of the three biological replications. Means labeled with different lowercase letters indicate significant differences (*p* < 0.05) between the control and GA-coated group for every 15 days according to Duncan's multiple range tests.

Several studies have shown that postharvest film coating improves the activities of SOD, CAT, POD, and APX to reduce the oxidative damage that is caused by excess ROS accumulation in harvested horticultural fruits (9, 21, 26, 41). Herein, enzymatic antioxidants, and the results of SOD, CAT, POD, and APX activities are shown in Figure 3. The SOD activity decreased gradually as the storage days increased. However, a decrease in the SOD activity was notably (*p* < 0.05) postponed by the GA coating treatment during the last 60 days of the storage period. As compared to the initial SOD activity (40.6 U g^−1^), SOD activity losses in the 9, 12, and 15% GA-coated Ponkan fruit were 38.8, 26.6, and 33.3% at the end of storage whereas the loss in the control fruit was 47.8% (Figure 3A). The CAT activity exhibited an increasing trend at an early stage of storage and peaked at 45 days of cold storage, followed by a continuous decline (Figure 3B). The level of CAT activity in the 12% GA-coated Ponkan fruit was much higher in comparison with the control and 9% GA-coated group during the middle to end period of cold storage (from 45 to 120 days). In 45 days, the highest CAT activity (88.3 U g^−1^) was exhibited in the 12% GA-coated fruit, followed by 15% GA coating (81.0 U g^−1^), 9% GA coating (74.5 U g^−1^), and the lowest CAT activity (72.3 U g^−1^) was recorded in the control fruit, indicating that the peak of CAT activity in the 12% GA-coated fruit was much higher in comparison with that in control and other two GA-coated groups. As shown in Figure 3C, the changes of POD activity in Ponkan fruit displayed similar trends as that of SOD activity. After 45 days of cold storage, Ponkan fruit treated with 9, 12, and 15% GA coating maintained a higher overall POD activity by 9.4, 36.0, and 25.3%, respectively, compared to the control fruit. The APX activity in the control and 9% GA-coated Ponkan fruit increased rapidly and reached its peak at 45 days, followed by a sharp drop (Figure 3D). The APX activity in the 12 and 15% GA-coated Ponkan fruit peaked at 60 days, a delay of 15 days in comparison with the control. In the last 60 days of cold storage, the lowest level of APX activity was recorded in the uncoated control group, whereas the highest level was revealed by Ponkan fruits subjected to 12% GA-coated treatment.

In response to postharvest senescence stress, the non-enzymatic antioxidant amounts cooperated with an antioxidant enzyme system in the fruit tissue are of great importance to maintain cell structural integrity and reduce postharvest oxidative damage (4, 8, 40, 44). In plant cells, higher non-enzymatic antioxidant levels and antioxidant enzyme activities are critical for scavenging excessive ROS (e.g., ${\text{O}}_{2}^{•-}$, ^1^O_2_, •OH, and H_2_O_2_) that is generated in different subcellular compartments, including mitochondria, chloroplasts, peroxisomes, cytoplasm, and endoplasmic reticulum (12, 32). With the dismutation of ${\text{O}}_{2}^{•-}$ to H_2_O_2_, SOD plays its predominant role in ROS scavenging; subsequently, the dismutation-generated H_2_O_2_ was directly catalyzed into H_2_O with the concerted effort by CAT, POD, and APX (11, 19). In our current study, the pre-storage treatment with 12% GA coating prominently increased both CAT (Figure 3B) and APX activities (Figure 3D), suppressed the decline of SOD (Figure 3A) as well as POD activities (Figure 3C), and enhanced the elimination of ROS capacity of harvested Ponkan fruit *via* the improvement of SOD, CAT, POD, and APX activities. The 12% GA-coated fruit retained a greater level of antioxidant enzymes, including SOD, CAT, POD, and APX, and exhibited less oxidative damage in Ponkan fruit during cold storage at 10°C. Nie et al. (9) reported a delay in postharvest senescence in 1.5% chitosan-coated pummelo (*Citrus grandis* L. Osbeck) fruit may be ascribed to higher levels of ROS-scavenging enzyme activities, giving rise to lower electrolyte leakage and MDA accumulation, thereby protecting the integrity of the cell membrane and reducing juice sac granulation. Simultaneously, the maintained cell structural integrity in GA-coated “Grand Nain” bananas may be ascribed to lower electrolyte leakage arising from more POD and PPO activities (24). More SOD, CAT, POD, and APX activities are responsible for the reduction of postharvest decay and for the maintenance of cell membrane integrity as mirrored by lower MDA accumulation, which postponed senescence in the 12% GA-coated Ponkan fruit during 120 days of cold storage.

**Figure 4:**
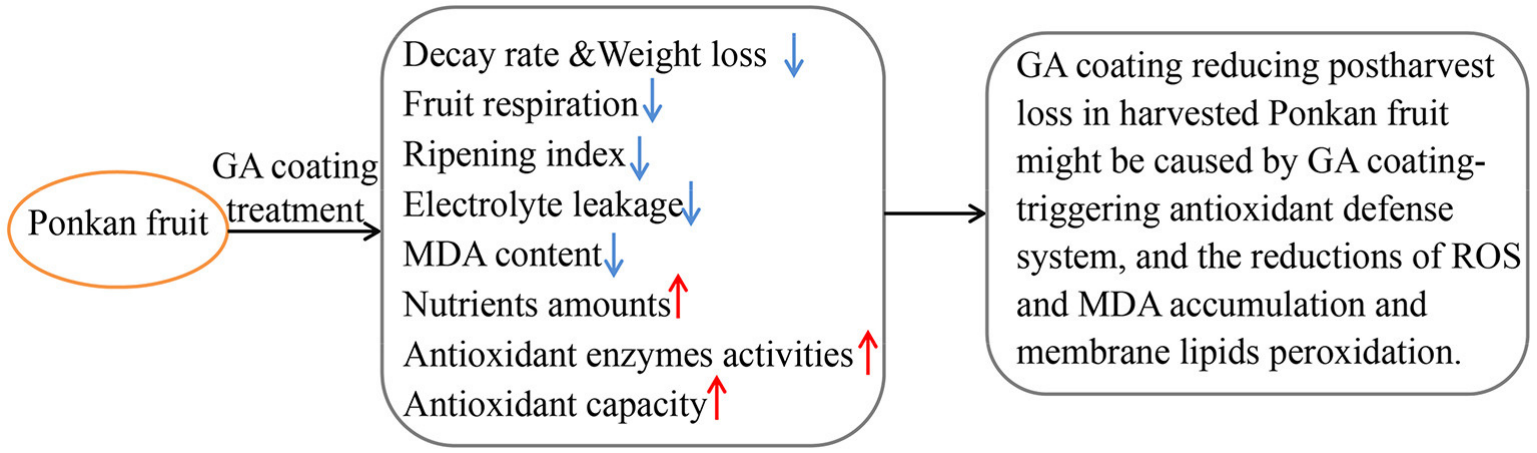
A probable mechanism of GA coating preserving the postharvest nutritional quality of Ponkan fruit through the activation of an antioxidant defense system.

All the results demonstrated that postharvest decay and nutritional quality deterioration of Ponkan fruit was reduced as a result of 12% GA coating for citrus fruit preservation. The possible mechanism of GA coating that preserves the postharvest nutritional quality of Ponkan fruit through the activation of an antioxidant defense system is shown in Figure 4.

## Conclusion

In brief, the postharvest decay and nutritional quality deterioration of fresh Ponkan fruit were closely related to the imbalance of ROS metabolism. Our work reveals the preservative effect of GA coating in regulating ROS metabolism to reduce postharvest decay and maintain the nutritional quality of harvested Ponkan fruit during cold storage. Specifically, the pre-storage treatment of 12% GA coating suppressed fruit respiration, lowered electrolyte leakage, delayed MDA accumulation, and triggered an antioxidant defense system, as shown by higher amounts of non-enzymatic component (e.g., AsA, phenols, and flavonoids) and more activities of ROS-scavenging enzymes, such as SOD, CAT, POD, and APX. These results showed that 12% GA coating effectively reduced postharvest loss (decay and weight loss) and retarded fruit quality deterioration because GA coating could maintain the antioxidant capacity (the amounts of non-enzymatic components and the activities of antioxidant enzymes) to reduce oxidative damage (electrolyte leakage) as well as to inhibit membrane lipid peroxidation (MDA content) in Ponkan fruit. Overall, this study suggested that the pre-storage application of GA coating treatment could be used as a prospective preservative to reduce the postharvest decay of Ponkan fruit and alleviate its nutritional quality deterioration during cold storage at 10°C for 120 days.

## Data Availability Statement

The raw data supporting the conclusions of this article will be made available by the authors, without undue reservation.

## Author Contributions

CC designed this experiment and wrote the original manuscript. QH performed the experiments. YZ analyzed the data. CW participated in the experiment of antioxidant activity and revised the manuscript. JC supplied a platform for the experiments. All authors approved the final revision of the manuscript for publication.

## Funding

The present study was supported by the Modern Agricultural Industry Technology System and Advantage Innovation Team Project of Jiangxi province, China (Grant No. JXARS-07 and 20181BCB24005) and Jiangxi 2011 Collaborative Innovation Center of Postharvest Key Technology and Quality Safety of Fruits and Vegetables (Grant No. JXGS-05).

## Conflict of Interest

The authors declare that the research was conducted in the absence of any commercial or financial relationships that could be construed as a potential conflict of interest.

## Publisher's Note

All claims expressed in this article are solely those of the authors and do not necessarily represent those of their affiliated organizations, or those of the publisher, the editors and the reviewers. Any product that may be evaluated in this article, or claim that may be made by its manufacturer, is not guaranteed or endorsed by the publisher.
